# Chronic and Asymptomatic Diffuse Alveolar Haemorrhage with Microscopic Polyangiitis: A Case Report and Review of the Literature

**DOI:** 10.1155/2016/1658126

**Published:** 2016-12-05

**Authors:** Hiroki Tashiro, Koichiro Takahashi, Hironori Sadamatsu, Masaru Uchida, Shinya Kimura, Naoko Sueoka-Aragane

**Affiliations:** ^1^Division of Haematology, Respiratory Medicine and Oncology, Department of Internal Medicine, Faculty of Medicine, Saga University, 5-1-1 Nabeshima, Saga 849-8501, Japan; ^2^Department of Internal Medicine, Karatsu Red Cross Hospital, 2430 Watada, Karatsu-shi, Saga 847-8588, Japan

## Abstract

Diffuse alveolar haemorrhage (DAH) is one of the major causes of death in microscopic polyangiitis (MPA) patients, because of acute respiratory failure with various respiratory symptoms. We, herein, present a case of chronic and asymptomatic DAH in a patient with MPA who was diagnosed by fibreoptic bronchoscopy. The patient showed localized reticular shadows, without any respiratory symptoms, and absence of inflammatory reactions, such as fever and CRP elevation, which is atypical for DAH. Three months after appearance of the lung abnormalities, DAH with MPA was diagnosed by fibreoptic bronchoscopy. She was initially treated with only corticosteroids and has thereafter been maintained with corticosteroids and azathioprine without relapse to date. We reviewed the literature for similar cases and opined that physicians should perform fibreoptic bronchoscopy in MPA patients with chronic lung abnormalities and anaemia to identify DAH, even if the patients show no respiratory symptoms and in the absence of inflammatory reactions.

## 1. Introduction

Anti-neutrophil cytoplasmic antibody- (ANCA-) associated vasculitis (AAV) is a group of heterogeneous diseases, including microscopic polyangiitis (MPA), granulomatosis with polyangiitis (GPA), eosinophilic granulomatosis with polyangiitis (EGPA), and renal-limited AAV [1]. Diffuse alveolar haemorrhage (DAH), one of the major lung involvements in MPA patients, induces acute respiratory failure and is, therefore, associated with a poor prognosis in these patients [2, 3]. In general, most DAH patients have various respiratory symptoms, such as breathlessness, cough, haemoptysis, dyspnoea, and chest pain [4]. Herein, we report a case of chronic and asymptomatic DAH in a patient with MPA. In addition, we reviewed the reported cases of chronic progressive and asymptomatic DAH in patients with AAV.

## 2. Case Presentation

A 78-year-old Japanese woman was found to have anaemia and reticular shadows on her chest X-ray during a health examination, for which she came to our hospital. She did not have any symptoms, including fever, cough, and purulent sputum, at that time. Her dietary habits were normal and she had not experienced any weight loss. Her haemogram and serum biochemistry revealed the following: haemoglobin: 7.8 (11.3–15.2) g/dL, MCV: 78.5 (79–100) fL, MCHC: 29.5 (30.7–36.6)%, serum iron: 62 (43–172) *μ*g/dL, ferritin: 67.2 (4.6–204.0) ng/dL, unsaturated iron-binding capacity: 188 (137–327) *μ*g/dL, vitamin B12: 470 (180–914) pg/mL, folic acid: 3.8 (3.1–9.7) ng/mL, blood urea nitrogen: 20.9 (8–22) mg/dL, creatinine: 1.13 (0.4–0.7) mg/dL, sodium: 145 (138–146) mEq/L, potassium: 3.5 (3.6–4.9) mEq/L, and chlorides: 109 (99–109) mEq/L. Her faecal occult blood test was negative. We diagnosed the cause of anaemia as defective iron utilization, and we followed up her laboratory data and chest X-ray findings to clarify the cause of defective iron utilization.

Three months later, she was admitted to the hospital with renal failure, chest radiograph abnormality, and severe anaemia. She had a past medical history of hypertension but had never smoked and had no history of tuberculosis (TB). She had not been exposed to any fine particles. She did not have fever or any respiratory symptoms, such as breathlessness, cough, haemoptysis, dyspnoea, and chest pain.

**Figure 1:**
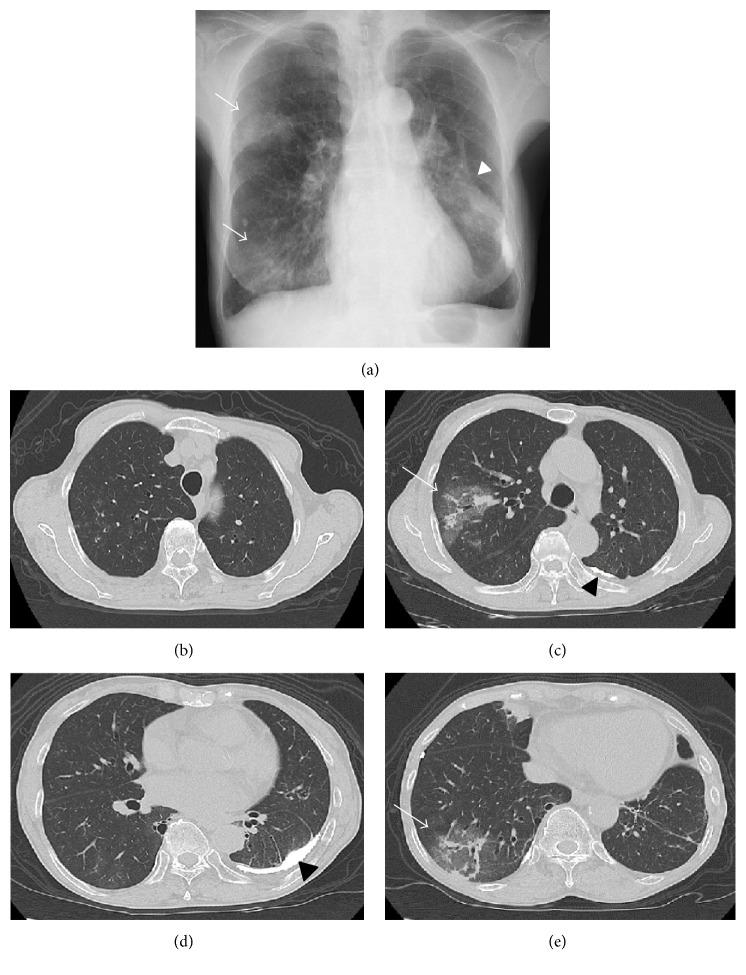
Findings on chest radiography and computed tomography. (a) Chest radiograph showing reticular shadows in the right middle and lower lung fields (arrow). Calcified lesions were seen in the left lung field (arrowhead). (b–e) Chest computed tomography (CT) showing segmental ground glass shadows in the upper and lower lobes of the right lung (arrow). The left pleura shows thickening with calcification (arrowhead).

Physical examination showed a body temperature of 36.3°C and no crackles were audible on auscultation. Her chest X-ray showed persistence of the reticular shadows in the right middle and lower lung fields. In addition, a calcified lesion was observed in the left middle part (Figure 1(a)). Chest computed tomography (CT) showed a ground glass shadow and consolidation in the right upper and lower lobes with left sided pleural thickening and calcification (Figures 1(b)–1(e)). Her laboratory findings at this time were haemoglobin 6.9 g/dL, white blood cell count 6,600 (3,500–9,000)/*μ*L, blood urea nitrogen 61.5 mg/dL, creatinine 2.56 mg/dL, C-reactive protein (CRP) 0.0 (<0.3) mg/dL, erythrocyte sedimentation rate (ESR) 90 (2–15) mm/h, myeloperoxidase (MPO)-ANCA 54 (<10) EU, proteinase 3 (PR3)-ANCA <10 (<10) EU, and anti-glomerular basement membrane (GBM) antibody <10 (<10) EU. More than 100 counts per high power field of red blood cell (RBC) casts were found on urinalysis.

**Figure 2:**
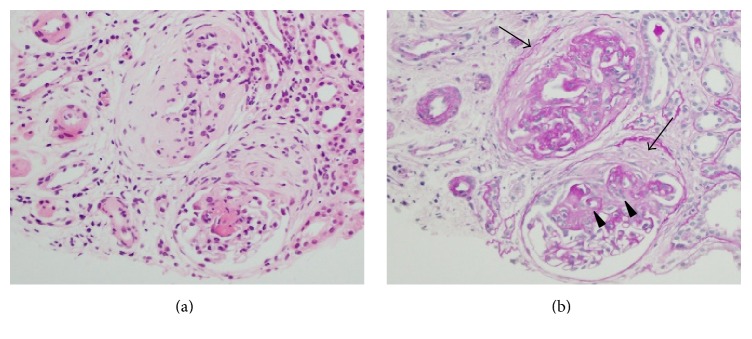
Pathological findings of the renal biopsy specimen. Cellular crescent formations (arrow) and lobulation were observed in the glomeruli (arrowhead) by (a) Haematoxylin and Eosin stain (×400) and (b) Periodic acid-Schiff stain (×400).

**Figure 3:**
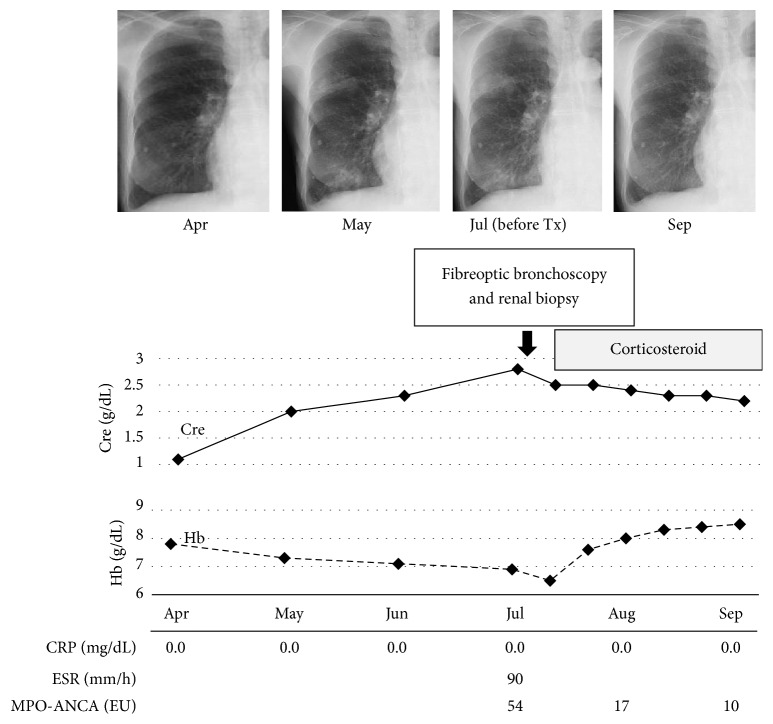
Clinical course of the MPA patient. Serial chest radiographs showed the clinical course of the pulmonary shadows in the right middle and lower lung fields. The solid line in the graph shows serum creatinine levels and the dashed line shows haemoglobin levels during the patient's clinical course. Tx: treatment.

Renal biopsy was performed and pathological evaluation of the specimen revealed cellular crescent formations and lobulation in more than half the glomeruli (Figure 2). Evaluation of bronchoalveolar lavage fluid (BALF) revealed increased RBC counts, and cytology indicated that 90% of the detected macrophages were haemosiderin-laden. These findings indicated the presence of alveolar haemorrhage and, hence, she was diagnosed as MPA. She did not consent to undergo surgical lung biopsy at this time. We initially planned to treat her with corticosteroids and intravenous cyclophosphamide (IVCY), and, therefore, we first started treatment with corticosteroid therapy (prednisolone 50 mg/day) (Figure 3). After prednisolone therapy at this dose for 14 days, we started tapering it by 10 mg every 2 weeks, and once the dose of 20 mg was reached we tapered it by 5 mg every 4 weeks. This resulted in improvement in the renal failure, anaemia and lung abnormalities and, hence, IVCY was not required. Once the corticosteroid dose was reduced to 5 mg, it was combined with azathioprine. Currently, the patient is on maintenance therapy with corticosteroids (prednisolone 3 mg/day) and azathioprine (5 mg/day) without relapse of lung and renal lesions. She has been carefully followed up for development of TB by examination of chest X-rays once a month and sputum culture every three months, but with no TB prophylaxis.

## 3. Discussion

MPA is pathologically characterized by inflammation and fibrinoid necrosis of small vessel walls, leading to multiple organ involvement, including of the kidneys, lung, skin, digestive system, and nervous system. The aetiology of AAV is considered to involve genetic and environmental factors, such as silica exposure, infections, and drugs [8]. The pathogenesis of AAV is known to be related to both innate and adaptive immune systems. The complement system and neutrophils play major roles in the effector phase of the pathogenic immune response in AAV. Specifically, T helper (Th) cells have been noted to be involved and recently, Th17 cells were reported to contribute to production of proinflammatory cytokines (IL-1*β* and TNF-*α*), which results in the priming of neutrophils [8]. These mechanisms suggest that the pathogenesis and activity of AAV is strongly related to inflammation.

DAH is a common and clinically important complication in MPA patients. Typical chest radiological patterns of DAH show focal or diffuse areas of ground glass opacification and/or consolidation [9]. However, these findings are nonspecific, and other aetiologies, such as lung infections, and interstitial pneumonia, show similar radiographic abnormalities. Fibreoptic bronchoscopy, particularly with BALF evaluation, is the best method to specifically diagnose DAH and for excluding other diseases [10]. Some studies of DAH patients with AAV showed that almost all patients have respiratory symptoms, such as dyspnoea and haemoptysis [3, 11, 12]. Additionally, DAH progresses rapidly and causes acute respiratory failure, such that the patients sometimes require admission to the Intensive Care Unit (ICU). Cartin-Ceba et al. reported that 34 of 73 patients with DAH secondary to AAV (47%) underwent mechanical ventilation (MV), and 41 patients (56%) were admitted to the ICU [11]. Moreover, DAH was reported to be an independent prognostic factor of MPA and the mortality rate of MPA patients with DAH is 8.65 times greater than that of MPA patients without DAH [5]. These data suggest that DAH induces various respiratory symptoms and that the development of acute respiratory failure in MPA patients is associated with high disease activity.

Although our patient did not have any respiratory symptoms, we believe that the alveolar haemorrhage in the present case might have existed for more than 3 months before diagnosis, because the shadow in her right lung had gradually progressed over 3 months without any features of pneumonia or interstitial lung disease. However, the DAH was finally diagnosed by performing fibreoptic bronchoscopy and was successfully treated with corticosteroids. Three similar cases of chronic and asymptomatic DAH with MPA have been previously reported [6, 7, 13]. A review of the four cases, including the present case, is presented in Table 1. In these cases, MPO-ANCA was the most detected antibody and anti-glomerular basement membrane (GBM) antibody was detected in one case. All the cases had severe but unidentified anaemia, suggesting chronic progression of anaemia. All three previous cases, but not our case, had a prolonged interval between DAH onset and diagnosis. Our patient, on the other hand, was diagnosed at an early stage, since we promptly performed fibreoptic bronchoscopy to identify the aetiology of the lung abnormalities, and we were, thus, able to make a definitive diagnosis from among DAH, lung infections, and interstitial pneumonia. Furthermore, detection of the DAH also led to a definite diagnosis of MPA in our case. Our experience suggests that physicians should aggressively perform fibreoptic bronchoscopy to identify the aetiology of lung disorders, even if patients do not have any respiratory symptoms or acute progressive lung findings. Furthermore, we believe that demonstrable anaemia is one of the clinical findings of chronic and asymptomatic DAH.

Interestingly, almost all four cases did not have inflammatory reactions, such as elevation of fever and serum CRP. However, the present case and the patient described in [6] had elevated ESR levels. ESR tends to show false positive results in patients with anaemia and renal failure. A previous large scale study indicated that CRP levels are a better biomarker of inflammation than ESR and ESR is frequently a misleading biomarker which shows false positive [14]. The present case and the patient in [6] both had severe anaemia and renal failure, which could have resulted in false positive ESR levels. Regarding the relationship between the disease activity of AAV and biomarkers, Kronbichler et al. reported that CRP titre was one of the important factors of ANCA-associated disease activity [15]. Moreover, DAH patients with MPA without acute respiratory failure often demonstrate significantly lower CRP levels than patients with acute respiratory failure [11]. These data suggest that there may be rare phenotypes of DAH with MPA, which manifest with less inflammation and lower disease activity.

Although the present case did not have an obvious medical history of TB, her X-ray showed pleural calcification, indicating the possibility of resolved TB. Immunosuppressive therapy and abnormal chest X-ray findings are reported to be associated with a high risk for development of active TB [16]. We did not administer therapy for TB to our patient in consideration of its adverse effects. Instead, we followed her up carefully for the development of TB by frequent chest X-ray and sputum culture examinations.

In conclusion, we report a case of DAH with MPA that progressed chronically with no respiratory symptoms. Physicians should perform fibreoptic bronchoscopy in MPA patients with lung abnormalities and unidentified anaemia as soon as they can, even if the patients have no respiratory symptoms or elevation of CRP.

## Figures and Tables

**Table 1:** Clinical characteristics of cases of chronically progressive DAH with MPA reported in the literature, including the present case.

Ref. number	Age	Gender (M/F)	Detected antibody	Duration at the onset of DAH	Respiratory symptoms	Complications	inflammatory reaction such as fever, CRP titre
[5]	61	F	Anti-GBM antibody, ANCA	2 years	Breathless	Renal failure, anaemia (Hb 6.0 g/dL)	N.A.
[6]	23	F	MPO-ANCA	4 years	No	Renal failure, anaemia (Hb 6.3 g/dL)	no fever, normal CRP titre, 80 mm/h of ESR
[7]	11	F	MPO-ANCA	1 year	No	anaemia (Hb 5.9 g/dL)	no fever
Present case	78	F	MPO-ANCA	More than 3 months	No	Renal failure, anaemia (Hb 6.9 g/dL)	no fever, normal CRP titre

GBM: glomerular basement, ANCA: anti-neutrophil cytoplasmic antibody, MPO-ANCA: myeloperoxidase anti-neutrophil antibody, Hb: haemoglobin, CRP: C-reactive protein, and ESR: erythrocyte sedimentation rate.
